# Gene Expression Profiling Analysis of Bisphenol A-Induced Perturbation in Biological Processes in ER-Negative HEK293 Cells

**DOI:** 10.1371/journal.pone.0098635

**Published:** 2014-06-05

**Authors:** Rong Yin, Liang Gu, Min Li, Cizhong Jiang, Tongcheng Cao, Xiaobai Zhang

**Affiliations:** 1 Department of Chemistry, Tongji University, Shanghai, China; 2 Shanghai Key Laboratory of Signaling and Disease Research, the School of Life Sciences and Technology, Tongji University, Shanghai, China; Sun Yat-sen University, China

## Abstract

Bisphenol A (BPA) is an environmental endocrine disruptor which has been detected in human bodies. Many studies have implied that BPA exposure is harmful to human health. Previous studies mainly focused on BPA effects on estrogen receptor (ER)-positive cells. Genome-wide impacts of BPA on gene expression in ER-negative cells is unclear. In this study, we performed RNA-seq to characterize BPA-induced cellular and molecular impacts on ER-negative HEK293 cells. The microscopic observation showed that low-dose BPA exposure did not affect cell viability and morphology. Gene expression profiling analysis identified a list of differentially expressed genes in response to BPA exposure in HEK293 cells. These genes were involved in variable important biological processes including ion transport, cysteine metabolic process, apoptosis, DNA damage repair, etc. Notably, BPA up-regulated the expression of ERCC5 encoding a DNA endonuclease for nucleotide-excision repair. Further electrochemical experiment showed that BPA induced significant DNA damage in ER-positive MCF-7 cells but not in ER-negative HEK293 cells. Collectively, our study revealed that ER-negative HEK293 cells employed mechanisms in response to BPA exposure different from ER-positive cells.

## Introduction

Bisphenol A (BPA) is an important industrial chemical mainly used as an intermediate in the manufacture of polycarbonate plastics and epoxy resin. BPA has become ubiquitous in the environment due to the extensive use of BPA-containing products including food and beverage packaging, flame retardants, adhesives, building materials, electronic components, and paper coatings. Human bodies are often exposed to BPA that leaches out of containers especially under high temperature and acidic conditions [1], [2]. Large-scale surveys have shown that more than 90% of the study population has detectable levels of BPA in urine [3], [4], [5].

Due to the ubiquity of BPA exposure, more and more attention has been paid to the potential health effects induced by BPA [6], [7]. BPA exhibits estrogenic properties, and has been identified as a classical endocrine disrupting chemical that can affect the endocrine system through mimicking or disrupting endogenous estrogens [7], [8]. Epidemiologic studies and animal studies showed that BPA exposure contributed to numerous female reproductive disorders, and also suggested that pregnant women, fetuses, infants and children may be most vulnerable to the effects of BPA exposure [9], [10], [11]. BPA was first declared a toxic substance excluded from infant formula bottles in Canada in 2010, and then was banned in infant formula bottles in European Union in 2011. Besides the impacts on reproductive system and development, exposure to BPA has been associated with several chronic diseases such as cardiovascular disease, diabetes, liver disease and cancers [5], [12], [13].

Previous studies on health effects of BPA exposure mainly relied on animal models and epidemiological surveys [14], [15], [16], [17]. While these observations indicate that BPA exposure is potentially harmful to human health, validation of the findings in human remains challenging due to several reasons. For epidemiological studies, there is virtually no unexposed population due to the ubiquity of BPA [2]. The half-life of BPA is short, and the impacts of BPA exposure on human health usually take a long time to emerge. Thus, it is difficult to determine the causal links between BPA exposure and harmful health effects, especially chronic diseases. In vitro experiments were conducted to reveal the direct impacts of BPA exposure on cell viability and gene expression. Due to the estrogen-like properties of BPA, these studies mainly focused on BPA impacts on individual genes of interest in estrogen receptor (ER)-positive cells [18], [19], [20]. The genome-wide impacts of BPA exposure on gene expression especially in ER-negative cells is yet to be uncovered. To characterize the cellular and molecular effects of BPA on ER-negative cells, we performed RNA-seq to examine perturbation on gene expression exerted by low-dose BPA in HEK293 cells. We did not observe changes in cell morphology and viability. Gene expression profiling analysis identified a list of differentially expressed genes with variable functions. Interestingly, there are on common genes between the differentially expressed genes in ER-negative HEK293 cells and those in ER-positive cells. Particularly, BPA caused DNA damage in MCF-7 cells but not in HEK293 cells. Taken together, BPA affected gene expression in ER-negative HEK293 cells in a manner different from that in ER-positive cells.

## Materials and Methods

### Cell Culture and BPA Treatment

The human embryonic kidney 293 cells (HEK293) were cultured at 37°C in 5% CO_2_ as adherent monolayer in Dulbecco modified Eagle medium (DMEM) (Hiclone) supplemented with L-glutamine and 10% fetal bovine serum (FBS) (Hiclone). BPA powder was dissolved in the dimethyl sulfoxide (DMSO) and added to culture medium. The final concentration of BPA and DMSO is 10^−6^ M and 10^−3^ M, respectively. For BPA treatment, cells were treated with 10^−6^ M BPA for 48 h. Meanwhile, cells cultured in BPA-free medium were used as the control.

### RNA-seq Experiment

Total RNA was extracted from each sample using Trizol reagent (Invitrogen) according to the manufacturer’s instructions. mRNA enrichment, library preparation and sequencing were performed at BGI-Shenzhen (sequencing service provider). 49 bp single-end reads were generated for each sample on Illumina HiSeq2000 platform. RNA-seq data can be accessed through ArrayExpress database (www.ebi.ac.uk/arrayexpress/) under accession number E-MTAB-1959.

### Electrochemical Experiment for DNA Damage Detection

After removal of medium, cells were washed in PBS pH 7.4 and then smashed into fractions. DNA damage was detected by using catalytic oxidation with Ru(bpy)_2_dppz^2+^. Electrochemical measurements were performed with a CHI660C electrochemical workstation (CH Instruments, Inc., USA). The set-up of the electrochemical system was a conventional three-electrode system consisting of Ru(bpy)_2_dppz^2+^ modified glassy carbon electrode (GCE) as working electrode (GCE diameter: 3 mm), a saturated calomel electrode (SCE) as the reference electrode and a platinum wire as the auxiliary electrode. All volumetric flasks, beakers, pipettes, and other glassware were closely washed with chromosulfuric acid to remove possible contamination. Differential pulse voltammetry (DPV) was carried out to detect DNA damage from 0.0 to 0.6 V with pulse amplitude of 5 mV.

### Bioinformatics Analysis

RNA-seq reads were aligned to the human genome (hg19) using TopHat version 1.3.3 [21]. The reference genome and the corresponding annotation files were downloaded from the UCSC genome browser. Reads alignment was carried out with default parameters except for the following options: “-a 6 -m 2 -i 50–no-novel-juncs”. Only the uniquely mapped reads were used for the subsequent analysis. Reads distribution relative to gene structure was statistically analyzed using Ever-seq (http://code.google.com/p/ever-seq/). BEDTools [22] was used to calculate the coverage of reads along transcripts for each sample.

Cuffdiff, a separate program from Cufflinks [23], was used to calculate gene expression levels and test the statistical significance of expression changes between two samples. Reads Per Kilo base per Million mapped reads (RPKM) was used to evaluate gene expression levels. Pearson correlation coefficient was calculated to measure the overall similarity between transcriptome profiles of the BPA-treated sample and the control sample. Expression changes were measured by log2(fold change) with false discovery rate (FDR)-adjusted p-value as an indicator for statistical significance. Genes with |fold change|> = 1.5 and FDR-adjusted p-value< = 0.05 were defined as the differentially expressed genes as a consequence of BPA exposure.

To investigate the functions and involving pathways of genes affected by BPA exposure, we used Ingenuity Pathway Analysis software (IPA, http://www.ingenuity.com) to perform Gene Ontology (GO) and pathway analysis. To assess the biological relationships among these genes, we built molecular interaction networks among them.

## Results and Discussion

### Effects of BPA Exposure on Cell Growth and Gene Expression in HEK293 Cells

**Figure 1:**
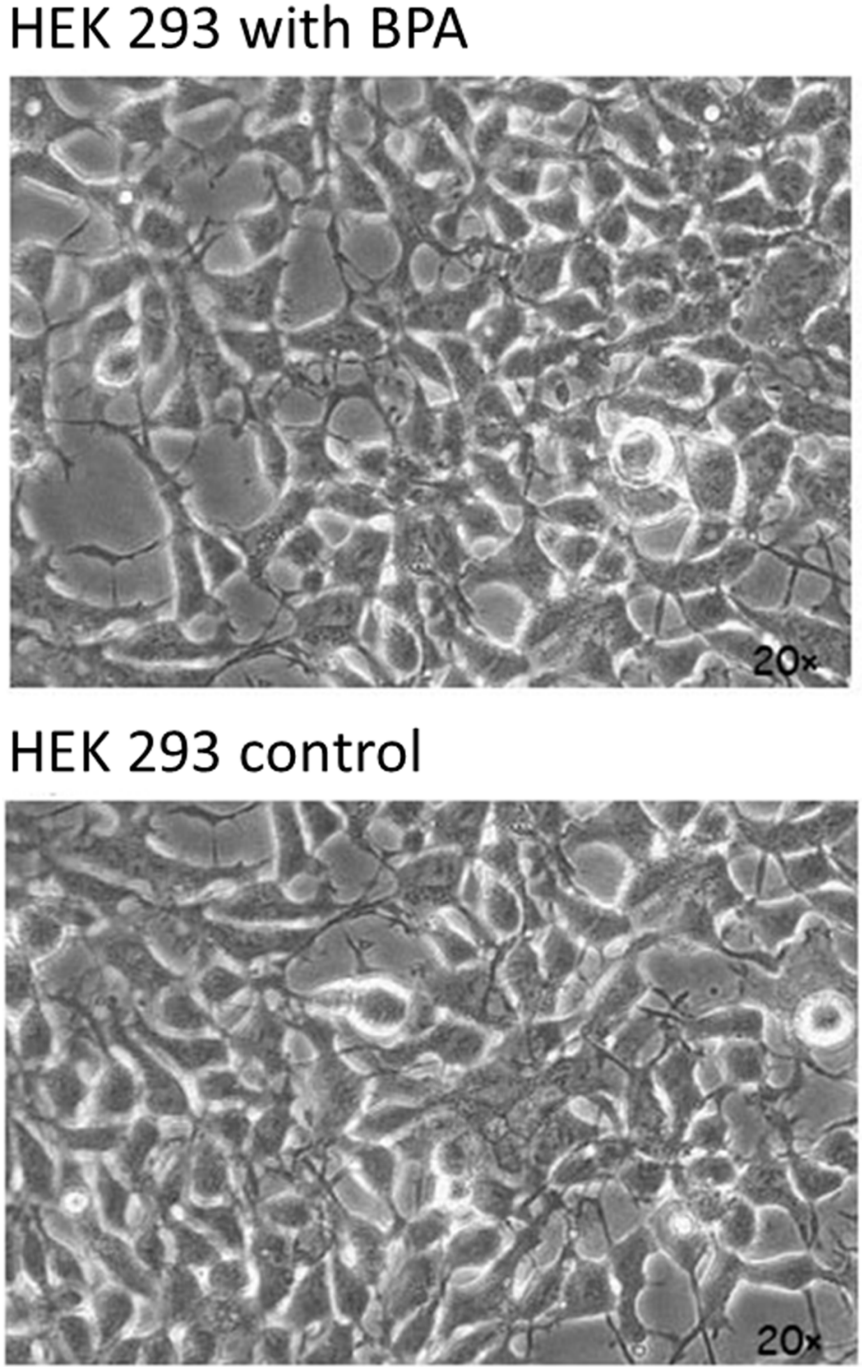
Microscope images showing morphology of HEK293 cells with and without BPA exposure. There are no observable changes in cell morphology of the two samples.

To assess cellular and molecular effects induced by BPA exposure of environmental relevant concentration, we investigated the toxicity of low-dose (10^−6^ M) BPA exposure on HEK293 cells. After 48 h treatment, we examined the effects of BPA exposure on cell morphology and transcriptome profile. The physiological status and morphology of cells treated with BPA are indistinguishable from those in the control sample (Fig. 1). This is consistent with the observations from previous studies suggesting that BPA exposure do not cause observable effects on viability of ER-negative cells [20], [24]. However, BPA exposure decreased cell survival through apoptosis induction in ER-positive MCF-7 cells and ovarian granulosa cells [25], [26].

In order to understand the molecular mechanisms by which ER-negative HEK293 cells response to BPA exposure at a dose that is environmentally pertinent. We systematically examined the gene expression changes induced by BPA treatment. High throughput sequencing technology was employed to generate the gene expression profiles for the BPA-treated sample and the control sample, and produce 8,243,449 and 8,547,485 clean reads, respectively (Table 1). More than 93% of these reads from both samples were mapped to the reference genome, and ∼85% of the reads were unambiguously mapped to a single location in the genome. These uniquely mapped reads were enriched in the coding regions and untranslational regions in both samples (Fig. S1). This indicated that these reads were from transcribed genes.

**Table 1:** RNA-seq read count in HEK293 cells with and without BPA treatment.

	BPA	Control
Total clean reads	8,243,449	8,547,485
Mapped reads (%)	7,684,990 (93.23%)	7,968,367 (93.22%)
Uniquely mapped reads (%)	6,953,073 (84.35%)	7,213,084 (84.39%)

**Figure 2:**
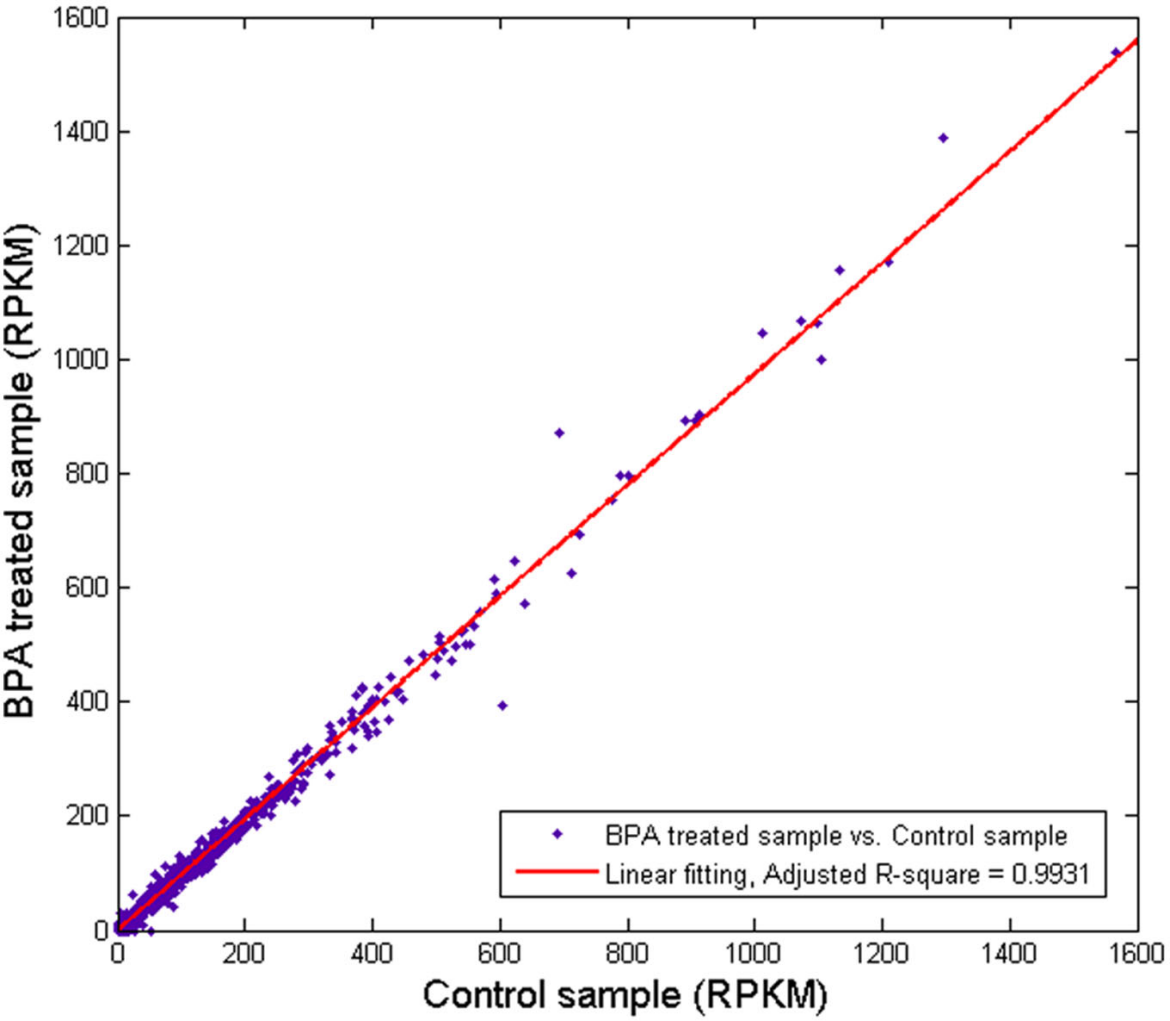
Comparison of gene expression profiles between BPA exposure and control samples. Linear fitting indicates highly similarity between the transcriptome profiles of the BPA-treated sample and the control sample.

We further analyzed the gene coverage by the reads for each sample and found that more than 60% of the annotated genes reached a coverage rate higher than 60%. This suggests that these uniquely mapped reads well represent expressed genes. Interestingly, both read distribution in the genomic regions and gene coverage in the two samples were similar to each other (Fig. S1 & S2). Moreover, the global expression profiles of the two samples showed strong similarity (Fig. 2). The comparison of two gene expression profiles identified 15 differentially expressed genes after BPA treatment, 8 up-regulated and 7 down-regulated, respectively (Table 2). We validated some of the differentially expressed genes using qRT-PCR (Fig. S3). This implied that BPA exposure led to a limited variation in gene expression in ER-negative HEK293 cells. This is likely attributed to the absence of ER in HEK293 cells. In contrast, BPA regulated ER target genes in ER-positive cells and could have more than 300 genes whose expression changed two or more folds [19].

**Table 2:** Significantly differentially expressed genes caused by BPA exposure and their annotated biological process GO terms.

Gene symbol	Description	Biological process	Regulation	Log2(fold change)	q-value
ERCC5	excision repair cross-complementing rodent repair deficiency, complementation group 5	nucleotide excision repair	Up	1.216	0.003
PTCD1	pentatricopeptide repeat domain 1	tRNA 3′-end processing	Up	1.046	0.001
SLC1A4	solute carrier family 1, member 4	glutamate receptor signaling	Up	0.752	0.002
CTH	cystathionase	cysteine metabolic process	Up	0.631	0.005
TRIM66	tripartite motif containing 66	transcription regulation	Up	0.629	0.036
BSN	bassoon presynaptic cytomatrix protein	cytoskeleton organization	Up	0.624	0.016
H6PD	hexose-6-phosphate dehydrogenase	pentose phosphate pathway	Up	0.623	0.005
PPP1R3E	protein phosphatase 1, regulatory subunit 3E	glycogen metabolic process	Up	0.619	0.039
HSPA8	heat shock 70 kDa protein 8	glucocorticoid receptor signaling	Down	−0.624	3.988e-7
BAX	BCL2-associated X protein	apoptosis signaling	Down	−0.635	0.037
PNO1	partner of NOB1 homolog	-	Down	−0.742	0.002
NOL9	nucleolar protein 9	phosphorylation; rRNA processing	Down	−0.753	4.125e-8
HSPA1B	heat shock 70 kDa protein 1B	glucocorticoid receptor signaling	Down	−1.132	0
ZNF460	zinc finger protein 460	regulation of transcription	Down	−1.277	0.029
AS3MT	arsenic methyltransferase	toxin metabolic process	Down	−2.019	0.021

### BPA Exposure Leads to Perturbation in Variable Biological Processes in HEK293 Cells

**Figure 3:**
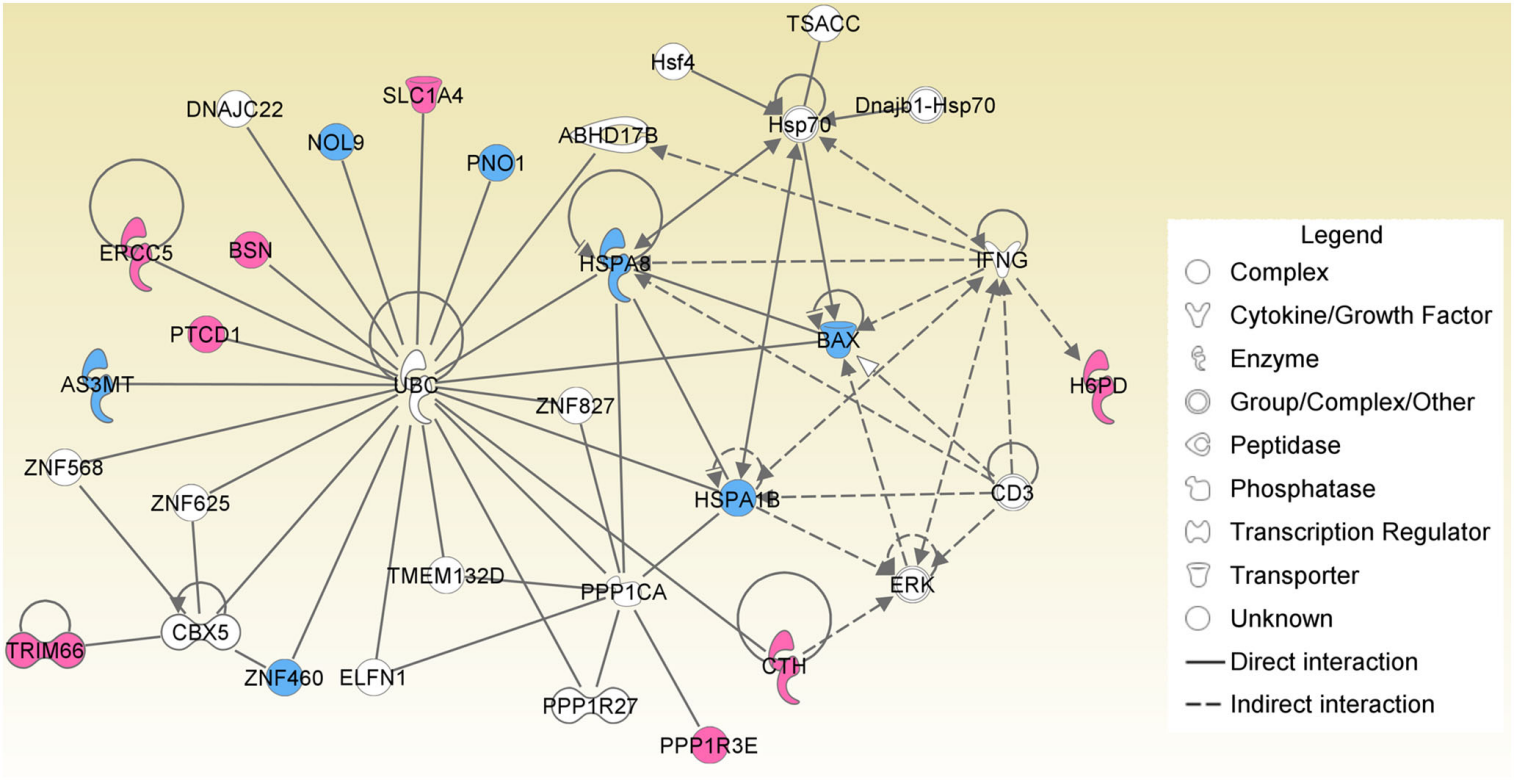
Interaction network of differentially expressed genes caused by BPA exposure. Genes are represented as nodes with various shapes for different types. Red and blue nodes indicate up-regulated and down-regulated genes induced by BPA, respectively. Connective edges represent various types of biological relationships among these genes.

IPA is a web-based functional analysis tool for identification of the most relevant signaling and metabolic pathways, molecular networks, and biological function for a list of genes. Here, we employed IPA to investigate the biological processes in which the differentially expressed genes involve. The results showed that BPA exposure perturbed many metabolic pathways including ion transport (SLCIA4), cysteine metabolic process (CTH, cystathionase), glycogen metabolic process, and toxin metabolic process (AS3MT, arsenic methyltransferase) (Fig. 3). CTH, a cytoplasmic enzyme in the trans-sulfuration pathway, converted cystathione derived from methionine into cysteine. Abnormal expression of CTH may cause cystathioninuria [27]. BPA exposure also influenced biological pathways including aldosterone signaling and glucocorticoid receptor signaling through down regulation of heat shock proteins HSPA8 and HSPA1B [28]. BAX, a pro-apoptotic regulator belonging to the BCL2 protein family, was down-regulated by BPA treatment. This suggests that low-dose BPA may reduce apoptosis of HEK293 cells. Additionally, BPA exposure altered expression of transcription factors TRIM66 and ZNF460, and regulated the expression of their target genes as a result. Notably, differentially expressed genes of BPA exposure include ERCC5 encoding a DNA endonuclease that involve in nucleotide-excision repair. ERCC5 was up regulated by BPA exposure. It will be interesting to further investigate whether up-regulation of ERCC5 is directly linked to the repair of DNA damage induced by BPA treatment in HEK293 cells. Although BPA mainly functions as an ER agonist and disrupts normal endocrine signaling through regulation of ER target genes, our results suggested that BPA had potential to influence variable physiological processes independent of ER. This may in part explain the facts that BPA exposure was associated with other chronic diseases such as cardiovascular disease, diabetes, liver disease and caners [5], [12], [13].

### BPA Causes DNA Damage in MCF-7 not in HEK293 Cells

**Figure 4:**
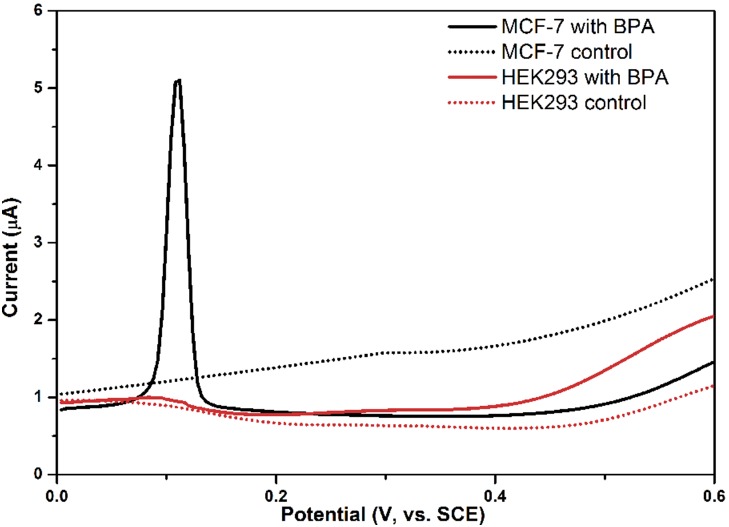
BPA induced significant DNA damage in ER-positive MCF-7 cells but not in ER-negative HEK293 cells. DPVs show voltammetric response to BPA-induced DNA damage in the range of 0.0 to 0.6 V (vs. SCE). The peak indicates DNA damage in MCF-7 cells. Solid lines indicate DPV signals in HEK293 cells (red) and MCF-7 cells (black) treated with 10^−6^ M BPA for 48 h, while dotted lines represent DPV signals in the corresponding control sample.

It has reported that BPA induced DNA damage in germ cells [16], [29]. Since ERCC5 encoding a DNA endonuclease was up regulated in HEK293 cells, it is unclear to what extent the increased expression level of ERCC5 can ameliorate DNA damage by BPA. Therefore, we further used electrochemical method to detect DNA damage in ER-negative HEK293 cells and ER-positive MCF-7 cells both with BPA treatment. Adenine and guanine bases in DNA undergo electrochemical oxidations when DNA is chemically damaged. Damaged DNA reacts more rapidly than intact ds-DNA with Ru(bpy)_2_dppz^2+^ [30]. Thus, DPV method is able to detect damaged DNA on GCE by using catalytic oxidation with Ru(bpy)_2_dppz^2+^. Under pH 7.4 condition, DPVs of DNA-damage were recorded as shown in Figure 4. An obvious oxidation peak was observed at 0.110 V (vs. SCE) in BPA-treated MCF-7 cells but not in HEK293 cells. The peak clearly indicated DNA damage in MCF-7 cells. The absence of the peak, i.e., no detectable DNA damage, is likely due to the up regulation of ERCC5 (Table 2) in HEK293 cells by BPA exposure that promoted DNA repair. Consistently, ERCC5 was not up regulated in the previous studies on ER-positive cells [18], [19]. Interestingly, a previous study reported that BPA caused DNA damage through estrogenic activity [31]. Thus, we speculated that BPA maybe induced DNA damage in a cell type-specific manner.

BPA functions as an ER agonist and regulates ER target genes. Roles of BPA in disrupting normal endocrine signaling have been studied in ER-positive MCF-7 cells. Two recent gene expression profiling studies in ER-positive cells with BPA treatment identified a list of genes differentially expressed in response to BPA exposure [18], [19]. Interestingly, comparison analysis found no common genes between the list of genes and those in our study. A study using endometrial cancer cell line ECC-1 and breast cancer cell line T-47D showed that BPA induced binding of estrogen receptor 1 to the target sites dependent of cell type [18]. BPA also altered expression of thyroid specific transcription factors in thyroid follicular cells [24]. Collectively, this suggested that impacts of BPA on regulation of gene expression was likely cell type-specific. It also implied that BPA could disrupt variable physiological processes in ER-negative cells, though it mainly disrupted normal endocrine signaling as an ER agonist.

## Supporting Information

Figure S1 **Distribution of uniquely mapped reads in genomic features.**
(TIFF)

Figure S2 **Distribution of gene coverage in BPA-treated and normal HEK293 cells.** Gene coverage is the proportion of a gene region covered by RNA-seq reads and binned with an interval of 10%. Each gene belongs to one of the bin.
(TIFF)

Figure S3 **qRT-PCR validation of differentially expressed genes.** There are three replicates for each gene. The p-value was from t-test.
(TIF)
