# Supplementary figures and images for: Gene Expression Profiling Analysis of Bisphenol A-Induced Perturbation in Biological Processes in ER-Negative HEK293 Cells
Source: PLoS One. 2014 Jun 5;9(6):e98635. doi: 10.1371/journal.pone.0098635 (PMC4047077; doi:10.1371/journal.pone.0098635)

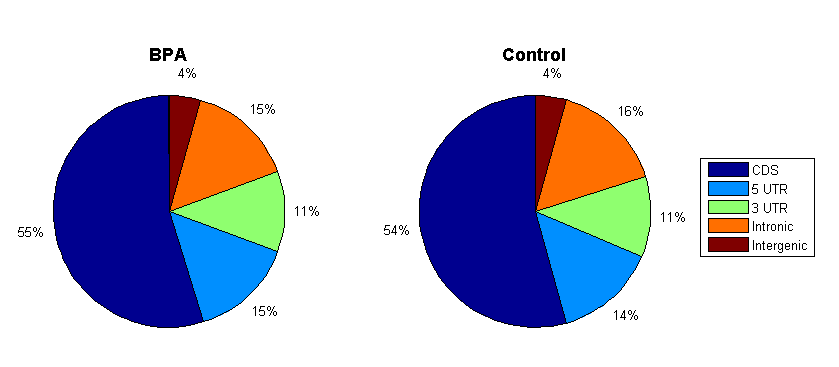

Supplement: Figure S1 — Distribution of uniquely mapped reads in genomic features. (TIFF) [file pone.0098635.s001.tif]

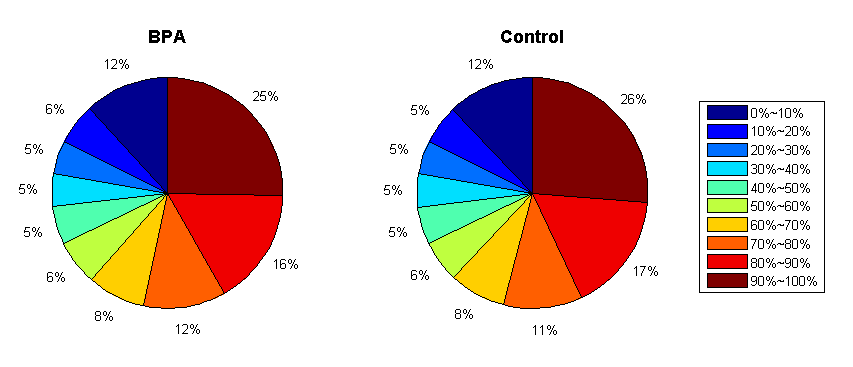

Supplement: Figure S2 — Distribution of gene coverage in BPA-treated and normal HEK293 cells. Gene coverage is the proportion of a gene region covered by RNA-seq reads and binned with an interval of 10%. Each gene belongs to one of the bin. (TIFF) [file pone.0098635.s002.tif]
